# Safety and efficacy of stereotactic radiofrequency ablation for very large (≥8 cm) primary and metastatic liver tumors

**DOI:** 10.1038/s41598-020-58383-y

**Published:** 2020-01-31

**Authors:** Peter Schullian, Edward W. Johnston, Daniel Putzer, Gernot Eberle, Gregor Laimer, Reto Bale

**Affiliations:** 1 From the Department of Radiology, Section of Interventional Oncology - Microinvasive Therapy (SIP), Medical University of Innsbruck, Anichstr. 35, 6020 Innsbruck, Austria; 2 From the Department of Radiology, King’s College Hospital, Denmark Hill, London, SE5 9RS UK

**Keywords:** Liver cancer, Surgical oncology

## Abstract

To assess the safety and clinical outcomes of multi-probe stereotactic radiofrequency ablation (SRFA) for very large (≥8 cm) primary and metastatic liver tumors with curative treatment intent. A retrospective, single center study carried out between 01.2005 and 06.2018. 34 consecutive patients had a total of 41 primary and metastatic liver tumors with a median size of 9.0 cm (8.0–18.0 cm) at initial SRFA. Patients were treated under CT guidance using a 3D navigation system. Endpoints consisted of (i) technical efficacy; primary - requiring one treatment, and secondary – requiring two treatments (ii) complication and mortality rates (iii) local and distant recurrence (LR), (iv) disease free survival (DFS), (v) overall survival (OS). 33/41 tumors were successfully ablated at initial SRFA (80.5% primary technical efficacy rate (PTE)). Four tumors required repeat ablation, resulting in a secondary technical efficacy (STE) rate of 90.2%. Local tumor recurrence (LR) developed in 4 of 41 tumors (9.8%). The 30-day perioperative mortality was 2.3% (1/ 44 ablations). The total major complication rate was 20.5% (9 of 44 ablations). Three of nine (33.3%) major complications, such as pleural effusion, pneumothoraces or perihepatic hemorrhages were relatively easy to treat. The overall survival (OS) rates at 1-, 3-, and 5- years from the date of the first SRFA were 87.1%, 71.8%, and 62.8% for patients with hepatocellular carcinoma (HCC) and 87.5%, 70.0% and 70.0% for patients with intrahepatic cholangiocarcinoma (ICC) respectively. Patients with metastatic disease had OS rates of 77.8% and 22.2% at 1- and 3- years. The clinical results of SRFA in this study are encouraging and warrant a prospective multicenter study. SRFA may become one of the best therapeutic choices for a growing number of patients with primary and metastatic liver cancer.

## Introduction

Radiofrequency (RF) ablation has been increasingly accepted as a curative and cost-effective treatment for small liver tumors^1,2^. Whilst hepatic resection (HR) is still the preferred treatment in patients with preserved liver function, in clinical practice less than one-third of the patients are eligible for HR at diagnosis^3,4^. Historically, the reported local recurrence rates after conventional RF ablation are relatively high and vary from 10% to 39.1% by 5 years, depending on tumor size and number^5,6^. It has been shown that an ablation zone ‘safety margin’ is required around tumors by at least 5 mm to achieve local control and good clinical outcome^7^. However, achieving adequate treatment margins may be challenging in large and irregularly shaped tumors or in situations where the target tumor is either difficult to visualize, awkward to access, or adjacent to vulnerable structures. Similar considerations apply to HR, with larger tumors more likely to be unresectable due to small future liver remnant and close relationship with the major vessels or hepatic hilum^8^.

Transarterial chemoembolization (TACE) is currently the recommended treatment method in patients with impaired liver function and large (>5 cm) hepatocellular carcinoma (HCC)^9,10^ and whilst a survival benefit has been demonstrated^10,11^, it is considered a palliative treatment^12^.

To overcome limitations in RF ablation zone size, other thermal ablation strategies have been developed including use of microwave (MW) energy and placement of multiple RF electrodes^13–16^. However, conventional multi-probe RF ablation using ultrasound or CT-fluoroscopy guidance lacks reliability for accurate three-dimensional RF probe placement and thus complete tumor coverage. Stereotaxy has proven useful for planning and executing complex or challenging ablations where access routes can be specifically facilitated and more precise coverage of the target tumor and safety margins can be accomplished^17,18^.

We have previously shown that SRFA is a viable treatment option in HCC^19^, although its role in very large lesions remains undefined.

The purpose of the current study was to assess the safety and clinical outcomes of multi-probe SRFA for very large (≥8 cm) primary and metastatic liver tumors, treated with curative intent. Specifically, our endpoints are primary (requiring one treatment) and secondary (requiring two treatments) technical efficacy, complication rates, recurrence, disease free and overall survival.

## Materials and Methods

### Selection criteria

The Institutional Review Board of the Medical University of Innsbruck approved this single-center, retrospective study. All participants gave their informed consent to collect their data. All treatment plans were consensually agreed in multidisciplinary tumor board meetings.

**Figure 1:**
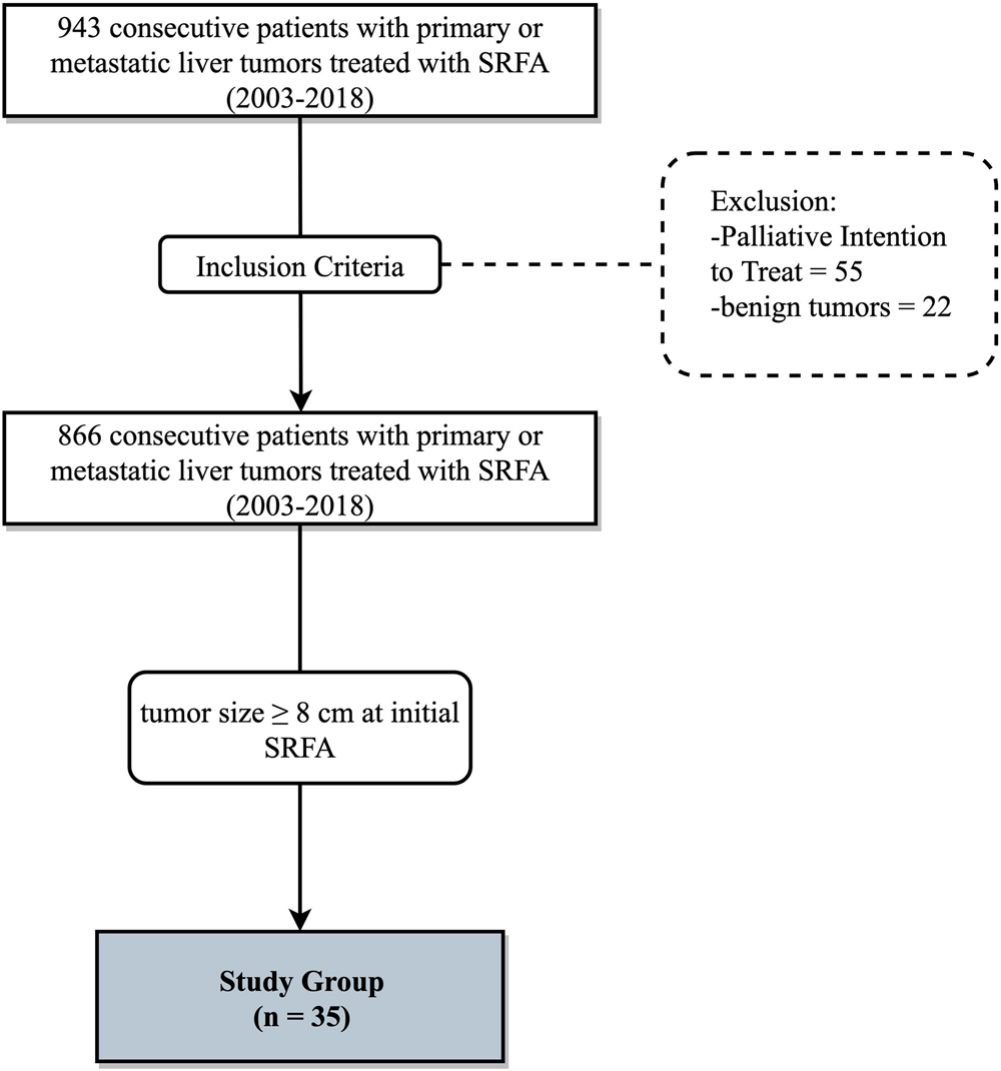
Flowchart of group assignment.

Between 01.2005 and 06.2018, 943 consecutive patients were treated by SRFA. Seventy-seven patients with portal venous invasion, extensive tumor with subsequent palliative intention to treat, or benign liver tumors were excluded (Fig. 1). Thirty-four patients with tumors ≥8 cm at initial SRFA were included. Baseline characteristics of included patients are shown in Table 1.

**Table 1:** Patient characteristics of 34 patients undergoing 44 SRFA sessions of 41 very large liver nodules.

Patient Characteristics	Study Group
Age, median years (range)	66 (27–81)
Sex (female/male), n (%)	7/27 (20.6/79.4)
Tumor Type, n (%)	
HCC, n (%)	16 (47.1)
ICC, n (%)	8 (23.5)
Metastasis, n (%)	10 (29.4)
Colorectal, n (%)	4 (11.8)
Breast, n (%)	2 (5.9)
Melanoma, n (%)	1 (2.9)
Other, n (%)	1 (2.9)
Cirrhosis, n (%)	12 (34.3)
Child A, n (%)	10 (22.2)
Child B, n (%)	2 (4.4)
Child C, n (%)	1 (0.2)
Tumor Size, median (range)	9.0 cm (8.0–18 cm)
Tumor Number at first SRFA, n (range)	1 (1–9)
n = 1, n (%)	22 (64.7)
n = 2, n (%)	7 (20.6)
n > 2, n (%)	5 (14.7)

SRFA = stereotactic radiofrequency ablation, HCC = hepatocellular carcinoma, ICC = intrahepatic cholangiocarcinoma, Child = Child Pugh Score.

A platelet count of <50000/mm^3^ and prothrombin activity <50% were exclusion criteria for SRFA. Tumor diagnosis was confirmed by typical imaging appearances on multiphasic contrast MRI or CT, with histopathological confirmation before or during SRFA procedure.

### Multi-probe stereotactic radiofrequency ablation - procedure

**Figure 2:**
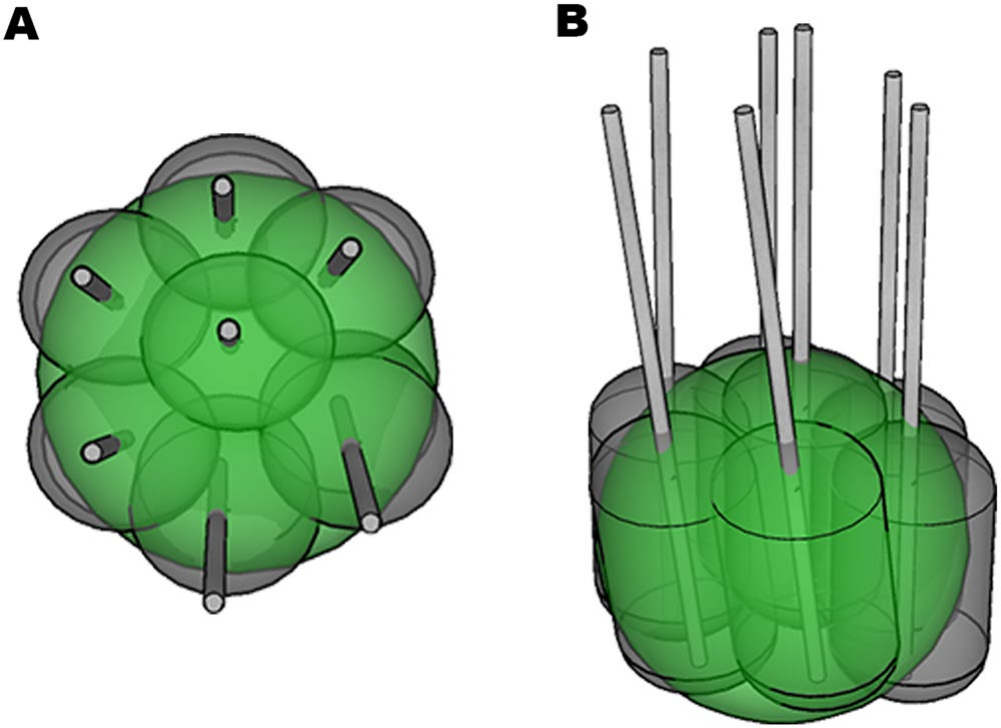
Illustration of multi-probe RF ablation with multiple overlapping coagulation zones (grey ellipsoids with central RF probe) covering the entire tumor volume (green sphere). (**A**) top view, (**B)** oblique view.

The basic principle of multi-probe RF ablation is the simultaneous usage of multiple RF probes to overcome ablation size limitation of single probe techniques by creating multiple overlapping ablation zones (Fig. 2). “Stereotaxy” derives from two Greek roots - “stereos” meaning solid, and “taksis” meaning arrangement. Stereotactic technique relates the position of targets and entrance points within the body to a Cartesian coordinate system.

The method of SRFA has previously been reported in detail^20–22^. In brief, the whole treatment is performed under general anesthesia with muscle paralysis. Patients are immobilized on the CT-table by a single (Bluebag, Medical Intelligence Schwabmünchen, Germany) or double vacuum fixation technique (BodyFix, Medical Intelligence Schwabmünchen, Germany). For image-to-patient registration, 10–15 fiducials, (X-SPOT, Beekley Corporation, Bristol, CT, USA) are broadly attached to the skin of the thorax and upper abdomen. A contrast-enhanced planning CT (Siemens SOMATOM Sensation Open, sliding gantry with 82 cm diameter, Siemens AG, Erlangen, Germany) is obtained with 3 mm slice thickness. To enable precise stereotactic conditions, the endotracheal tube (ETT) is temporarily disconnected during the planning CT, during each stereotactic needle placement and for the final control CT. The CT dataset is transferred to an optical-based navigation system (Stealth Station Treon plus, Medtronic Inc., Louisville, KY, USA). Multiple SRFA probe trajectories with an interprobe spacing of 1.5–2 cm are planned by using multiplanar and 3D reconstructed images.

After registration and a registration accuracy check, the probe of the navigation system is introduced into the ATLAS aiming device (Medical Intelligence Inc., Schwabmünchen, Germany). After alignment of the probe axis with the planned virtual trajectory by using the guidance software of the navigation system, 15G/17.2 cm coaxial needles (Bard Inc., Covington, GA, USA) are sequentially advanced through the adjusted targeting device without real-time imaging control. The depth from the aiming device to the target is automatically calculated by the navigation software. For verification of correct needle placement, a native control CT is performed and fused with the planning CT. In cases with lack of histological confirmation, a 16G coaxial biopsy sample is obtained. Thereafter, three 17G RF-electrodes (Cool-tip, Medtronic, Mansfield, MA, USA, 25 cm in length with 3 cm exposure) are introduced through the coaxial needles for serial tumor ablation. RF ablation is carried out using the unipolar Cool-tip RF generator (Cool-tip, Medtronic, Mansfield, MA, USA), including the Cool-tip_RF switching controller. The standard ablation time for three electrodes is 16 minutes. However, if a significant increase of impedance is observed (the so-called ‘roll-off effect’) the ablation process is finished. Track ablation is performed during every repositioning and final removal of the RF-electrodes to reduce bleeding and tumor seeding.

For verification of the ablation zone and for assessment of complications, a final contrast-enhanced CT scan is superimposed onto the planning CT.

**Figure 3:**
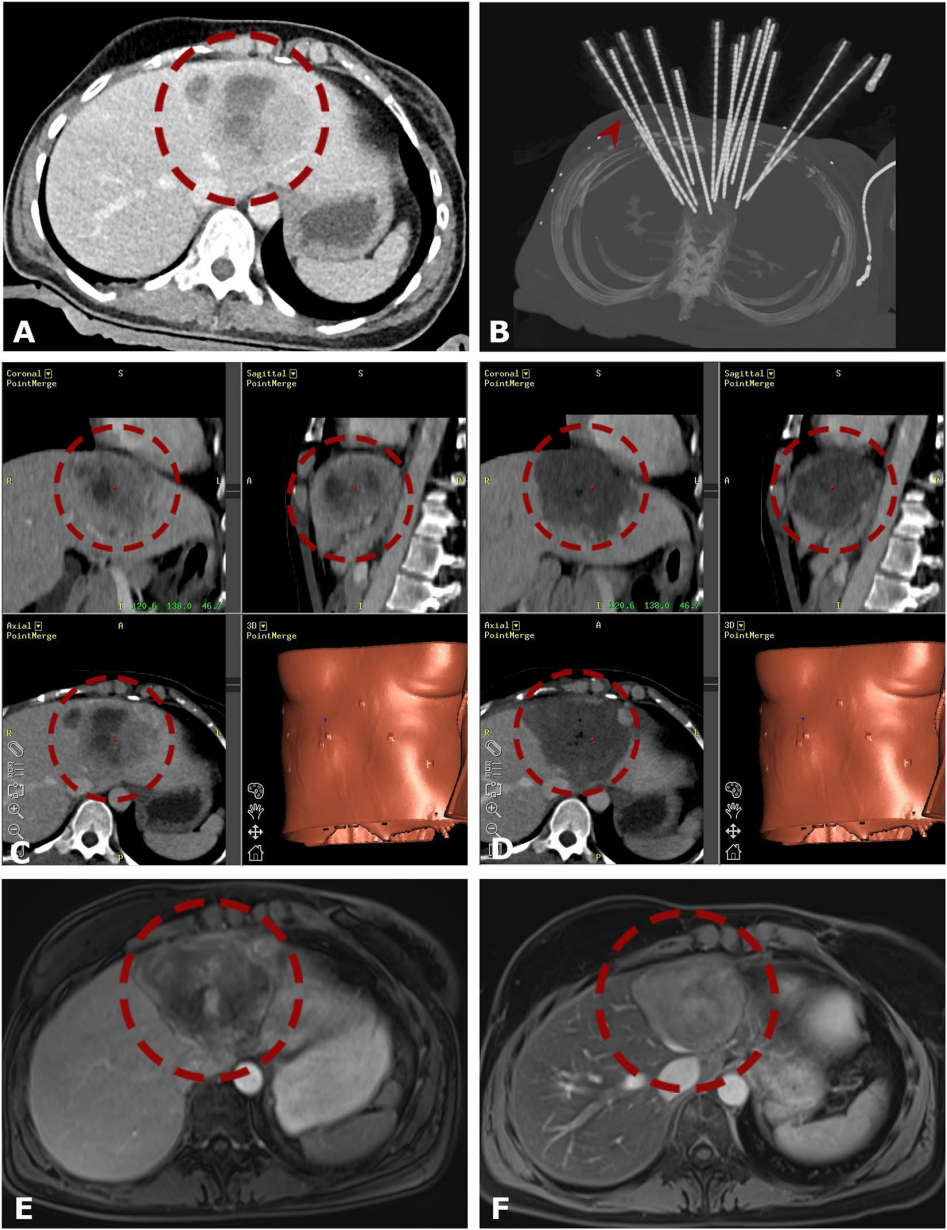
Case of a 40-year old female with a 9.0 cm breast cancer liver metastasis in segment IVa/II close to the heart. (**A**) Portal venous phase initial CT-scan with a hypo-enhancing large nodule in segment IVa/II (*red dashed circle*). (**B**) Maximum Intensity Projection (MIP) of the control CT with 15 coaxial needles in place (*red arrowhead*). (**C**,**D**) Fused CT-images of the contrast enhanced planning and final control CT showing a complete coverage of the tumor (*left image*) by the coagulation zone (*right image*). (**E,F**) The *red dashed circle* illustrates the progressively shrinking coagulation zone on MR images at 3 months (**F**) and 12 months (**G**) after SRFA with no evidence of local recurrence.

**Figure 4:**
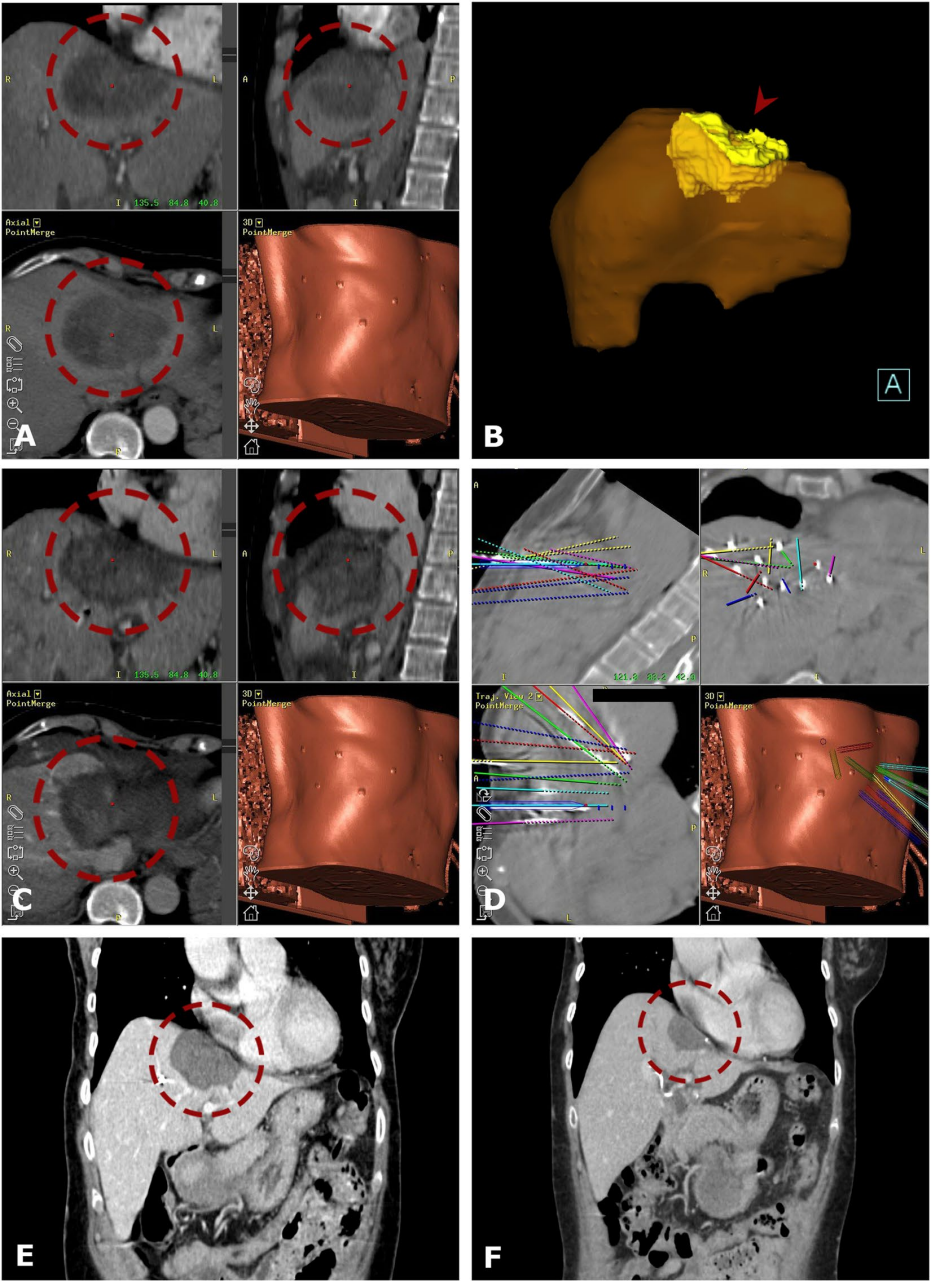
Case of a 62-year old female with a very large ICC IVa/II. (**A**) Fused CT-images of the ce-enhanced planning CT with a 9 cm HCC in segment IV/VIII (*red dashed circle*). (**B**) 3D volume rendering of the tumor (*red arrowhead*). (**C**) Fused images from the navigation system with 3D views from the contrast enhanced final control CT with complete coverage of the tumor. (**D**) 3D views from the navigation system with the coaxial needles in place fused with the predefined paths (*colored lines*). (**E,F**) The *red dashed circle* illustrates the progressively shrinking coagulation zone on CT images at 2 years (**E**) and 7 years (**F**) after SRFA with no evidence of local recurrence.

Example images from multi-probe SRFA of very large liver tumors are shown in Figs. 3 and 4.

### Study endpoints

The endpoints of the study consisted of (i) technical success and efficacy rates, (ii) complication and mortality rates, (iii) local and distant recurrence rates, (iv) DFS and (v) OS.

(I). Technical success was defined as accurate electrode placement, with a deviation <1 cm at the needle tip, according to predefined plans. Primary technical efficacy, requiring one treatment with complete ablation, and secondary technical efficacy, requiring more treatments for complete ablation, were determined by follow-up contrast-enhanced CT or MR scans performed at 1- month. The imaging tests were evaluated by two board certified abdominal radiologists by consensus.
(II). Considering the complexity of the procedures and for better comparison to surgery, the study endpoint major complications were defined according to the Clavien-Dindo classification (Grade III+)^23^. Mortality was defined as death within 30 days after SRFA treatment.
(III). Local recurrence was specified as appearance of new enhancing nodules (CT of MR scans at 3–6 months intervals) within or directly adjacent to the initially tumor-free ablation zone. New enhancing nodules distant to the ablation zone and or to the initial tumor location were specified as distant tumor recurrence.
(IV). (IV, V) Survival was calculated from the date of initial SRFA to the date of death attributable to malignancy or other causes (i.e., event) or to the most recent follow-up visit (i.e., censoring).

### Statistical analysis

The statistical analysis was performed with IBM SPSS version 24 (IBM, Armonk, New York). The Kolmogorov-Smirnov-Test, tested normality of distribution. Data were expressed as total numbers, median and range. Survival data (OS and DFS) were analyzed with the Kaplan Meier method. The X² test was used to determine differences between categorical variables, and the Mann-Whitney U test between independent continuous variables. A two-tailed p-value of <0.05 was considered as statistically significant.

### Ethical approval

All procedures performed in studies involving human participants were in accordance with the ethical standards of the institutional and/or national research committee and with the 1964 Helsinki declaration and its later amendments or comparable ethical standards.

### Informed consent

Informed consent was obtained from all individual participants included in the study.

### Consent for publication

Consent for publication was obtained for every individual person’s data included in the study.

## Results

### Patient characteristics

34 Patients, 7 females and 27 males, with a median age of 66 years (range 27–81) underwent 44 SRFA treatments of 41 very large primary or metastatic liver tumors Table 1. Diagnoses were 16 (47.1%) HCCs, 8 (23.5%) ICCs, and 10 (29.4%) metastatic tumors. The majority (4/10) of metastatic disease originated from colorectal cancer. The median size of the 41 nodules was 9.0 cm (8.0–18.0 cm). At treatment begin, 22 (64.7%) patients had a solitary liver tumor, 7 (20.6%) had two and 5 (14.7%) patients had more than two tumors. 12 (34.3%) patients had underlying cirrhosis (10 (22.2%) Child-Pugh A, 2 (4.4%) Child-Pugh B and 1 (0.2%) Child-Pugh C). 6 (17.6%) patients underwent chemotherapy, 2 (5.9%) patients underwent surgical resection, 4 (11.8%) patients underwent TACE and 2 (5.9%) patients conventional RF ablation prior to (not combined) SRFA. None of the patients received any additional loco(regional) therapy such as resection, TACE or SIRT.

The histopathological results from the biopsies obtained during SRFA (in 18 patients) were positive for malignancy in all cases.

### Perioperative complications

Major perioperative complications are presented in Table 2. One death was attributed to bleeding after SRFA of an 8 cm ICC (mortality rate 2.3% (1/44 ablations)). The total major complication rate was 20.5% (9 of 44 ablations).

**Table 2:** Details of major complications directly related to SRFA.

ID	Age	Sex	Prim.	Cirr.	T/S	mS/S	N/S	Complication	Therapy
1	75	male	ICC	—	1	8.0 cm	6	Major hemorrhage, MODS, death (18 days)	AG-coiling, ICU
2	58	male	HCC	+(C)	3	8.0 cm	21	Liver failure	Acute LTX
3	45	female	ICC	+(A)	1	11.0 cm	19	Transient liver failure	ICU
4	80	male	HCC	—	1	9.0 cm	11	Thermal damage of Bowel	Laparoscopic Surgery
5	55	male	HCC	+(A)	1	8.5 cm	9	Thermal damage of Gallbladder	Laparoscopic Surgery
6	77	female	CRC	—	2	8.0 cm	12	Liver Abscess	CT-drainage
7	58	male	HCC	+(A)	2	8.5 cm	15	Pleural Effusion	Thoracocentesis
8	66	female	MEL	—	1	11.0 cm	10	Pleural Effusion	Thoracocentesis
9	81	male	HCC	—	2	8.0 cm	15	Pleural Effusion	Thoracocentesis

Gr. = group, SRFA = stereotactic radiofrequency ablation, Prim. = primary tumor, T/S = tumors per session, mS/S = maximal tumor size per session, N/S = needles per session, HCC = hepatocellular carcinoma, ICC = intrahepatic cholangiocarcinoma, CRC = colorectal carcinoma, PCA = prostate cancer, MEL = melanoma, Cirr. = cirrhosis (Child Classification), AG = angiography.

One patient developed acute liver failure after treatment of 3 HCCs (largest tumor 8 cm) requiring salvage liver transplantation. Thermal injuries of the bowel (1 patient) and gallbladder (1patient) had to be surgically treated. One liver abscess required CT guided drainage. Other complications included transient liver failure (1 patient) and pleural effusions (3 patients) requiring thoracoenteses respectively. Additionally, two perihepatic hemorrhages and one pneumothorax were treated within the same session by embolization and chest drain which did not influence the postoperative course. 3/9 (33%) major complications were successfully treated by the interventional radiologist within the next days by placing a pleural drainage in case of major pleural effusions (3 patients).

The median hospital stay after the ablation was 7 days, ranging from 3–21 days. The median number of inserted coaxial needles per tumor was 12 (6–31).

### Technical Success

SRFA was successfully completed according to plan in all 41 tumors (technical success rate 100%) Table 3. 33/41 tumors were successfully ablated at initial SRFA (80.5% primary technical efficacy rate). Two very large tumors with 18 cm and 10 cm in diameter required three and two ablation sessions, respectively. All other tumors were treated in one session. After the first follow-up, 4 tumors were retreated, resulting in a secondary technical efficacy rate of 90.2%. 6–31 (median 12) RF electrodes were inserted in each tumor.

**Table 3:** Tumor based therapy success rates.

Rate	Study Group
Technical Success, n (%)	41/41 (100%)
Primary Technical Efficacy, n (%)	33/41 (80.5)
HCC, n (%)	15/17 (88.2)
ICC, n (%)	9/12 (75.0)
MET, n (%)	9/12 (75.0)
Secondary Technical Efficacy, n (%)	37/41 (90.3)
HCC, n (%)	15/17 (88.2)
ICC, n (%)	11/12 (91.7)
META, n (%)	11/12 (91.7)
Local Recurrence, n (%)	4/41 (9.8)
HCC, n (%)	1/17 (5.9)
ICC, n (%)	2/12 (16.7)
META, n (%)	1/12 (8.3)

HCC = hepatocellular carcinoma, CRC = colorectal carcinoma, MET = metastatic liver tumors.

A sub analysis based on tumor type (HCC, ICC and Metastasis) showed the highest PTE rates for HCC tumors with 88.2% (15/17) and the lowest for ICC with 75% (9/12). However, the differences between groups were non-significant (p = 0.607).

### Local recurrence rate and distant recurrence

During a median imaging follow-up of 9.4 months (range 1–133 months) local tumor recurrence developed in 4 of 41 tumors (9.8%, Table 4). Distant tumor recurrence in the liver was found in 24 patients (70.6%), and extrahepatic metastasis in 6 patients (17.6%), respectively. 19 (55.9%) patients received repeated SRFA for newly developed tumors. 15 patients (44.1%) developed untreatable tumor progression with multiple new nodules or extrahepatic spread.

**Table 4:** Unsuccessful local tumor control after SRFA.

ID	Age	Sex	Prim.	Cirr.	Size	Probes	Seg.	Prop.	Ablation Time	Pre – Th.	Out-come
1	66	female	MEL	—	11 cm	10	II, III, IV	v, bd	115 min	TAE	IA
2	62	male	HCC	+(B)	12 cm	9	VII, VIII	—	120 min	—	IA
3	66	male	HCC	—	10 cm	11	VI, VII	sc	300 min	—	IA
4	64	male	ICC	—	9 cm	15	IVa	—	125 min	—	IA
5	74	male	ICC	+(A)	8 cm	13	VI, VII, VIII	v, o	146 min	—	IA
6	79	male	PCA	—	8 cm	10	VII, VIII	v, sc	90 min	—	IA
7	77	female	CRC	—	8 cm	9	VIII	o	125 min	—	IA
8	45	female	ICC	—	11 cm	12	I, II, IV, VII	v, bd	80 min	—	IA
9	71	male	ICC	—	10 cm	18	I, V, VI, VII, VIII	v, bd	300 min	—	LR
10	56	male	CRC	—	13 cm	21	V, VI, VII, VIII	sc, c	252 min	—	LR
8	45	female	ICC	—	11 cm	19	I, II, IV, VII, VIII	v, bd, sp	110 min	SRFA	LR
11	73	male	HCC	—	8 cm	9	I, IVa, VII, VIII	v, c	100 min	TACE	LR

SRFA = stereotactic radiofrequency ablation, Prim. = primary tumor, HCC = hepatocellular carcinoma, ICC = intrahepatic cholangiocarcinoma, CRC = colorectal carcinoma, PCA prostate cancer, Cirr. = cirrhosis, Seg. = liver segment, Prop. = location properties, v = close to major vessel, sc = subcapsular, sp = subphrenic, o = close to organ, bd = close to central bile duct, c = conglomerate of nodules, Pre-Th. = unsuccessful pre-therapy, IA = incomplete ablation, LR = local recurrence.

A sub analysis based on tumor type (HCC, ICC and Metastasis) showed the lowest LR rates for HCC at 5.9% (1/16) and the highest for ICC at 16.7% (2/12). However, the differences between groups were non-significant (p = 0.600).

### Overall and disease-free survival

**Figure 5:**
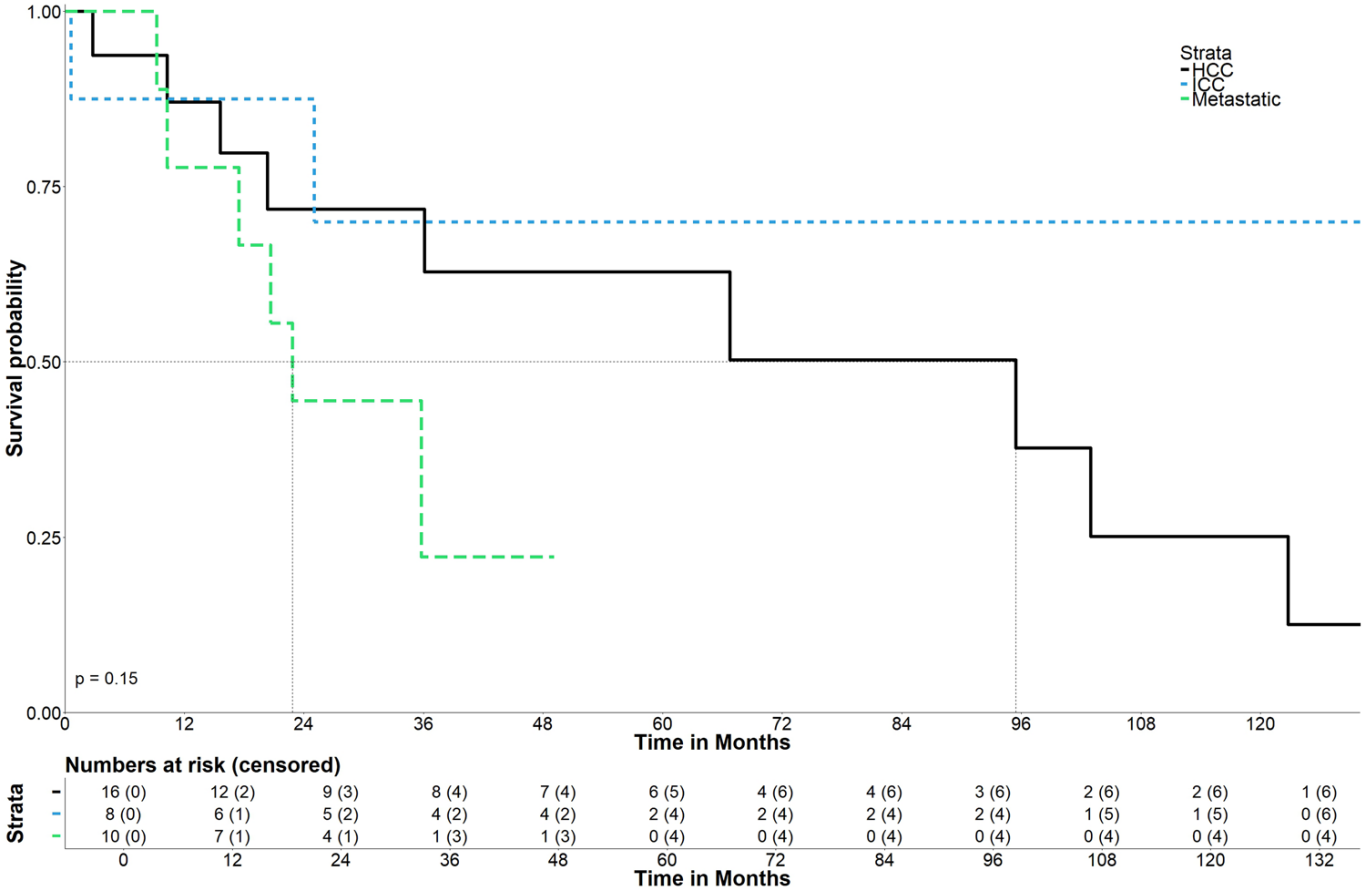
Overall survival after initial SRFA.

For HCC, the median OS and OS rates (Fig. 5) at 1-, 3-, and 5- years from the date of the first SRFA were 95.4 months (95% CI 36.1–169.4), 87.1%, 71.8%, and 62.8%, and for ICC 87.5%, 70.0% and 70.0%, respectively (median OS N/A).

Among patients with metastatic disease, the OS rates at 1- and 3- years were 77.8% and 22.2% with a median OS of 22.8 months (95% CI 17.4–36.2).

There was a difference, however, non-significant between groups based on tumor entity (p = 0.15).

**Figure 6:**
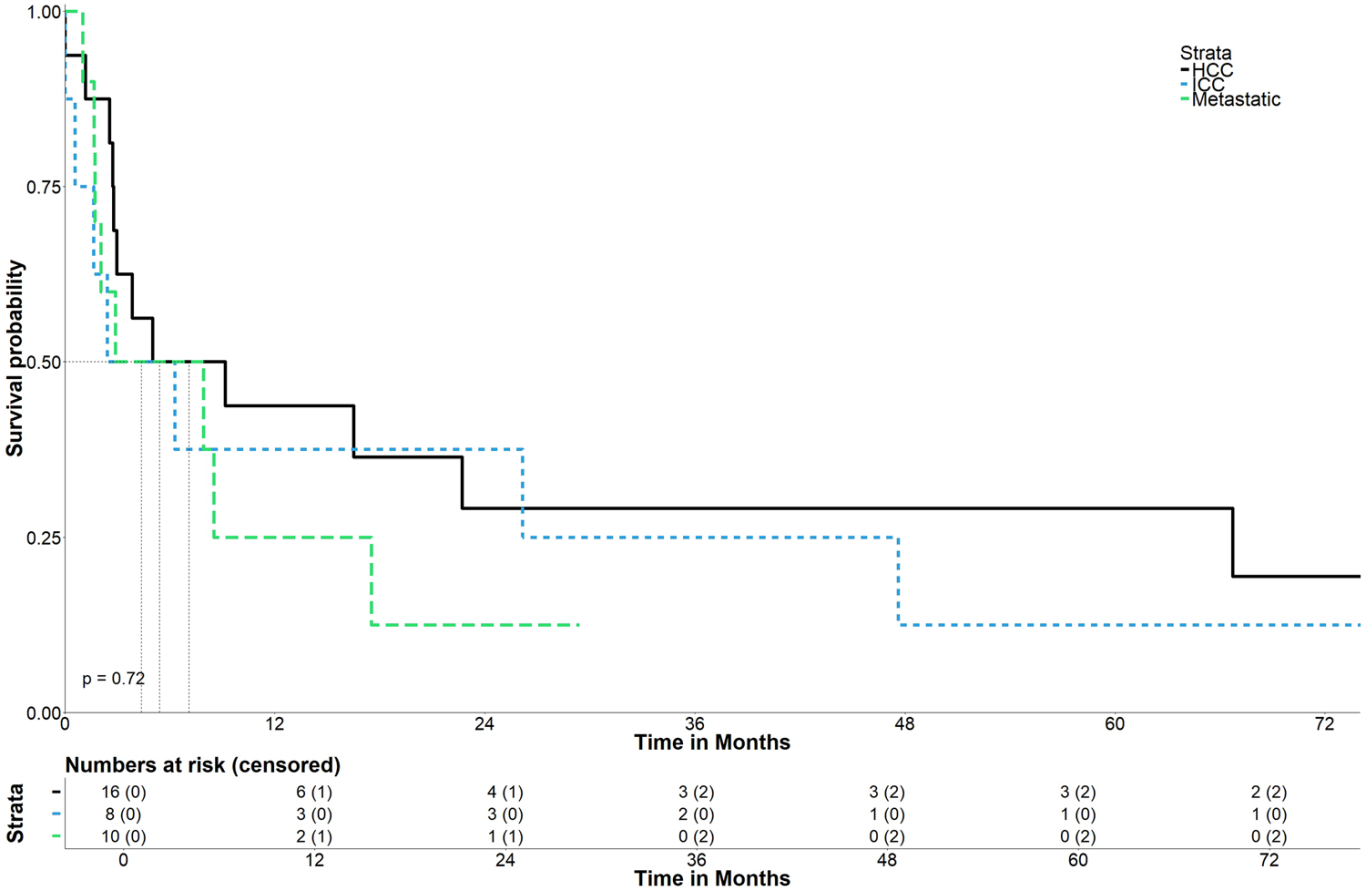
Disease-free survival after initial SRFA.

The disease-free (DFS) rates (Fig. 6) at 1-, 3-, and 5- years from the date of first SRFA were 43.8%, 29.2%, and 29.2%, 37.5%, 25.0% and 12.5% for patients with HCC or ICC with a median OS of 7.1 months (95% CI 0–15.5), and 4.35 months (95% CI 0–8.9), respectively. Among patients with metastatic disease, the DFS rates at 1- year were 25% with a median OS of 5.4 months (95% CI 0–16.8).

There was no significant difference between groups based on tumor entity (p = 0.72).

## Discussion

The main finding of our study is that SRFA is a feasible and effective treatment strategy for tumors >8 cm. To the best of our knowledge, there are no reports available dealing with thermal ablation of this tumor size, partially because HR remains the recommended treatment in large tumors with preserved liver function. However, the minority of patients are suitable for resection^3,4^, meaning other curative treatment options are required.

Since RFA results in complete cure for small HCCs (<3 cm) and colorectal liver metastases (CRLM) in greater than 90% of cases^24–26^, it has been incorporated into international guidelines for small tumors^12,27–29^. However, early studies demonstrated that complete cure falls to 40–70%^30,31^ for medium HCCs (up to 5 cm) and to 23–45% for large HCCs^31,32^ respectively, which is very likely to reflect the size limitations of RF ablation zones and the difficulties in producing overlapping ablations. To overcome size limitations, two main strategies were implemented. Firstly, increasing energy transfer in the form of microwave energy, and secondly using multiple RF probes to create overlapping ablation zones. More recent studies have consequently shown better PTE rates up to 86–97% in medium and large HCCs up to 10 cm^15,33,34^.

Whilst MWA can produce larger ablation zones faster and with less ‘heat-sink’ effect (i.e., large vessels reduce local tissue heating due to cooling) than RFA^35^, this higher thermal energy might injure adjacent vulnerable structures or create unnecessarily large ablation zones^36^. Whilst the lower energy transferred in RFA may result in more predictable ablation zones, the greater number of probes required considerably increases the complexity and difficulty of the procedure.

Accurate three-dimensional probe alignment to create sufficiently overlapping ablation zones demands a high level of operator experience using conventional US- and CT- guidance. However, SRFA offers three-dimensional ablation planning to achieve an optimal configuration of RF probes and create multiple overlapping coagulation volumes. Usage of an aiming device and triggering of respiratory motion^37^ facilitate precise path needle and probe placement^17^. Immediate post ablation contrast-enhanced CT fusion with the planning CT allows for rapid, reliable intraoperative judgment of the ablation results with the option for re-ablation within the same session. In a recent histopathological validation study in explanted livers after bridging therapy with SRFA, we found no viable tumor in 183 of 188 treated lesions (97.3%) and in 50 of 52 lesions >3 cm (96.2%).

Despite our inclusion of tumors up to 18 cm in diameter in the present study, LR developed in only 4 of 41 tumors (9.8%). In contrast, best results from the US- or CT- guided multiprobe thermal ablation literature with ‘freehand’ targeting quote LR rates of 15.9–19.7% after thermal ablation of large HCCs (largest tumors 10 cm, mean 3–5.7 cm) and other large liver tumors up to 8 cm (mean tumor size 2.8 cm)^33,34,38^.

OS rates in our study at 1-, 3-, and 5- years from first SRFA were 87.1%, 71.8%, 62.8%, and 87.5%, 70.0%, 70.0% for patients with HCC and ICC, respectively whereas patients with metastatic disease had OS rates of 77.8% and 22.2% at 1- and 3-years. Data are sparse regarding RF ablation of tumors >5 cm with Ma *et al*.^34^ reporting lower OS rates than in our study - specifically 65.5%, 36.3% and 21% at 1-, 3- and 5- years, respectively. Several retrospective studies compared HR and TACE in solitary HCC >5 cm, and reported 5-year OS of 43–65% and 17.5–49.6% for HR and TACE respectively^39–41^. Two studies regarding HR in HCC >5 cm by Zhao *et al*.^42^ and Hwang *et al*.^43^ reported OS of 75%, 59% and 44% and 78.4%, 58.8% and 49.3% at 1-, 3- and 5- years, respectively. For HCC (>10 cm), Zhou *et al*.^44^ showed OS of 60.7%, 34% and 28.6% at 1-, 3- and 5- years respectively. Data are sparse for HR of large CRLM with Nomit *et al*.^45^ reporting OS at 1-, 3- and 5- years of 87.2%, 56.1% and 35.4% respectively after laparoscopic HR.

In the present study, distant tumor recurrence was found in 70.6% of patients and median DFS rates were 4.4–7.1 months. These unfavourable prognostic indicators despite high levels of effective local tumor control (90.8%) might be explained by large lesions having a greater chance of vascular invasion^46^ and micrometastases^47^, and are line with the surgical literature at 57.1–82.4%^43,44^.

Our major complication rate of 20.5% is higher than in the study of Ma *et al*.^34^ and Laeseke *et al*.^32^ who reported a major complication rate of 16% following RFA for HCC >5 cm. One of the main reasons for that could be the high number of needles used (median 12 per tumor). However, our mortality rate compares favorably to Filmann *et al*.^48^ who reported a mortality of 9.1–25.5% after major HR in large outcome analysis of 110 332 procedures. Zhou *et al*.^44^ also published a systematic review after HR of large HCC (>10) a median perioperative morbidity of 29.2% (13.6–72%) and mortality of 3.5% (0–18.2%) and Nomit *et al*.^45^ reported a major complication rate of 50.0% after laparoscopic HR of large CRLM.

### Limitations

Study limitations include the retrospective design, heterogeneity of previous unsuccessful loco(regional) treatments (TACE or HR), and single treatment center bias. Comparisons with previous related studies are limited as stereotactic navigation systems were not employed in prior reports.

In summary, although all patients in this study were considered to be inoperable, we report lower mortality rates, lower complication rates and similar survival to the curative literature meaning SRFA may represent an important tool in the treatment of very large liver tumors.
